# An international multicenter retrospective analysis of patients with extranodal marginal zone lymphoma and histologically confirmed central nervous system and dural involvement

**DOI:** 10.1002/cam4.2732

**Published:** 2019-12-05

**Authors:** Andrew J. Sunderland, Raphael E. Steiner, Musa Al Zahrani, Chelsea C. Pinnix, Bouthaina Shbib Dabaja, Jillian R. Gunther, Loretta J. Nastoupil, Mats Jerkeman, David Joske, Gavin Cull, Tarec El‐Galaly, Diego Villa, Chan Yoon Cheah

**Affiliations:** ^1^ Department of Haematology Sir Charles Gairdner Hospital Nedlands WA Australia; ^2^ Department of Lymphoma/Myeloma MD Anderson Cancer Center Houston TX USA; ^3^ University of British Columbia and BC Cancer Centre for Lymphoid Cancer Vancouver British Columbia Canada; ^4^ Department of Medicine King Saud University Hospital Riyadh Saudi Arabia; ^5^ Department of Radiation Oncology MD Anderson Cancer Center Houston TX USA; ^6^ Skånes University Hospital Lund University Lund Sweden; ^7^ Department of Haematology Pathwest Laboratory Medicine WA Nedlands WA Australia; ^8^ Medical School University of Western Australia Crawley WA Australia; ^9^ Department of Hematology Aalborg University Hospital Aalborg Denmark

**Keywords:** CNS lymphoma, marginal zone lymphoma

## Abstract

Marginal zone lymphoma of the central nervous system (CNS MZL) is rare. The clinical features, treatment, and prognosis are not well characterized. We performed a multicenter retrospective study of CNS MZL. Twenty‐six patients were identified: half with primary and half with secondary CNS involvement. The median age was 59 years (range 26‐78), 62% female and 79% with ECOG performance status ≤ 1. The most common disease site was the dura (50%). Treatment was determined by the treating physician and varied substantially. After a median follow up of 1.9 years, the estimated 2‐year progression‐free (PFS) and overall survival (OS) rates were 59% and 80%, respectively. Secondary CNS MZL was associated with 2‐year OS of 58%. CNS MZL is rare, but relative to other forms of CNS lymphoma, outcomes appear favorable, particularly among the subset of patients with dural presentation and primary CNS presentation.

## Graphical abstract

Marginal zone lymphoma of the central nervous system (CNS MZL) is rare. In this multicenter retrospective study of 26 patients, the estimated 2‐year overall survival rate was 80%. Outcomes appear favorable, particularly among the subset of patients with primary dural presentation.

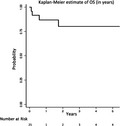

## 1 INTRODUCTION

The 2016 revision of World Health Organization classification of lymphoid neoplasms defined marginal zone lymphoma (MZL) as a non‐Hodgkin lymphoma (NHL) that includes three distinct subtypes: extranodal MZL of mucosa‐associated lymphoid tissue (MALT), nodal, and splenic MZL.^1^ Together, they account for approximately 10% of NHL and are the third most common histological subtype of NHL after diffuse large B‐cell lymphoma (DLBCL) and follicular lymphoma.^2^, ^3^ They are grouped together because they arise from postgerminal center marginal zone B cells and share histopathologic and immunophenotypic features.^4^, ^5^

MZL is considered an indolent lymphoma that typically occurs in association with autoimmune diseases and chronic inflammation and was first described by Isaacson and Wright as a low‐grade lymphoma of gastrointestinal tract MALT.^6^, ^7^ This is the most common site of origin where lymphoproliferation is associated with longstanding autoantigenic stimulation due to the action of *Helicobacter pylori* and other microbes.^3^, ^8^ MZL can originate from the mucosa of nongastrointestinal organs such as lung, bladder, salivary glands, conjunctiva, and lacrimal glands.^3^ Interestingly, MZL can also develop from tissue sites without mucosa including liver, breast, thyroid, orbit, skin, and the central nervous system (CNS).^3^, ^9^ Meningoepithelial cells of the arachnoid villi of dural venous sinuses could represent the cells of origin of CNS MZL where MALT‐type tissue develops secondary to chronic inflammation which provides a milieu for local accumulation and proliferation of antigen‐dependent B cells, leading to lymphoma development.^10^ Due to rarity, the clinical features and optimal management of CNS MZL are not well defined. To address this, we performed a retrospective study to describe the clinical characteristics, prognostic factors, treatment, and outcomes of patients with MZL and primary or secondary CNS involvement. We recognize that CNS MZL is truly defined by the presence of parenchymal and/ or leptomeningeal involvement, however, we have included patients with dural MZL in this analysis to describe the clinical features and response to treatment of this disease also.

## 2 METHODS

We searched institutional databases (MD Anderson Cancer Center; Sir Charles Gairdner Hospital; Aalborg University Hospital) and regional or national databases (BC Cancer Centre for Lymphoid Cancer database, Canada; Swedish Lymphoma Registry) to identify patients with CNS MZL who met the following inclusion criteria: (a) histopathologic diagnosis of MZL (b) histologically proven MZL of the dura, brain, or leptomeninges, or cytologically proven cerebrospinal fluid involvement at any time during disease course and (c) initial diagnosis of MZL between 1995 and 2018. Pathology review was performed by specialist hematopathologist at each listed center. Those with suspected DLBCL were excluded from analysis.

We collected baseline demographic and clinical data, treatment, and outcomes. Statistical analysis was performed using STATA 14.2 (STATA Corp). In patients with MZL and secondary CNS involvement, time to CNS involvement was calculated from date of systemic MZL diagnosis to date of confirmed CNS involvement. Median observation period was calculated from the median observation time of patients alive at last follow‐up. Overall survival (OS) was defined as time from CNS involvement to date of death with patients alive at last follow‐up censored. Progression‐free survival (PFS) was defined as the time of diagnosis of CNS disease to disease progression or death from any cause, with patients free from progression at that time point censored. Survival data were analyzed using the method of Kaplan‐Meier. Data collection was compliant with institutional requirements.

## 3 RESULTS

A total of 26 patients with histologically confirmed CNS MZL were identified. Clinical features at diagnosis with MZL and outcomes are summarized in Table 1. Half had primary CNS MZL at diagnosis, whereas the remaining half had secondary CNS MZL. Of the 13 patients with primary CNS MZL, 12 had biopsy proven MZL and for the remaining patient the specific method of detection was not documented. For the 13 patients with secondary CNS MZL, seven had biopsy proven MZL, whereas the remaining six had cytological evidence of MZL in CSF (3 of these had flow cytometric confirmation, for three patients the specific method of disease detection beyond cytology was not documented).

**Table 1:** Baseline characteristics

Features	Overall n = 26 (%)	Primary (n = 13)	Secondary (n = 13)
Age			
Median	59.5 y	51 y	62 y
Range	26‐78 y	26‐78 y	34‐77 y
Sex			
Female	16 (62)	9	7
Male	10 (38)	4	6
ECOG			
0	16 (62)	9	7
1	7 (27)	3	4
2	1 (4)	1	0
3	2 (8)	0	2
Stage at original MZL diagnosis			
I	12 (46)	11	1
II	2 (8)	1	1
III	0	0	0
IV	12 (46)	1	11
B symptoms			
Present	2 (8)	0	2
Absent	23 (88)	13	10
Missing	1 (4)	0	1
CNS location			
Dural only	13 (50)	9	4
Parenchymal only	5 (19)	3	2
Leptomeningeal only	1 (4)	0	1
Other	3 (12)	0	3
Missing	4 (15)	1	3
Serum LDH			
Normal	16 (62)	9	7
Elevated	7 (27)	3	4
Missing	3 (12)	1	2
Treatment			
RTx alone	1 (4)	0	1
CTx alone	5 (19)	0	5
SX + RTx	7 (27)	5	2
SX + CTx	6 (23)	4	2
RTx + CTx	0	0	0
SX + RTx +CTx	4 (15)	3	1
Missing	3 (12)	1	2
PF event			
No	13 (50)	11	2
Yes	10 (38)	2	8
Missing	3 (12)	0	3
Outcome			
Alive	22 (85)	13	9
Dead	4 (15)	0	4

Note

Data expressed as number (percentage).

Abbreviations: CTx, chemotherapy; LDH, lactate dehydrogenase; PF, progression free; RTx, radiotherapy; SX, surgery.

Of the 26 patients, 16 (62%) were female and the median age at diagnosis was 59.5 years (range of 26‐78). Most patients had good performance status at diagnosis (ECOG ≤ 1). All patients for which information was available presented with focal neurological symptoms including sensory loss, extremity weakness, visual changes, seizures and/or headaches. This is summarized in Table 2. Median duration of symptoms prior to CNS diagnosis was 105 days (range 30‐420) for primary disease and 60 days (range 0‐930 days) for secondary disease. Only two patients (8%) had B type symptoms at presentation and these patients had secondary CNS MZL stage IV disease with leptomeningeal involvement.

**Table 2:** Patient characteristics, treatment and outcome

No	Age/sex	Primary/Secondary	ECOG	LDH	BSx	Presentation	Site	Treatment	CTX Type	CNS Response	Status	OS (y)
1	38/F	Primary	0	Normal	No	Altered sensation bilateral upper limbs	C6 intramedullary cord	SX, CTx, RTx	MTX	CRu	Alive	1.4
2	78/F	Primary	1	Normal	No	Left hemiparesis	Right frontotemporal and parietal dura invading skull and temporalis muscle	SX, CTx	R‐CHOP + MTX	CRu	Alive	0.54
3	51/F	Primary	0	Unknown	No	Personality change	Left frontal dura	SX, CTx	R‐CHOP + MTX	CR	Alive	2.05
4	51/M	Primary	2	Normal	No	Unknown	Unknown	Unknown	N/A	CRu	Alive	2.41
5	26/M	Primary	0	Normal	No	Altered sensation face	Left medial temporal lobe dura	SX, CTx	R‐CHOP	CR	Alive	9.49
6	62/M	Primary	0	Normal	No	Seizure	Left fronto‐parietal region dura	SX, CTx, RTx	R	CR	Alive	3.84
7	54/F	Primary	0	Normal	No	Visual changes	Dura overlying tuberculum sella, suprasellar cistern, sella turcica	SX, RTx	N/A	CR	Alive	5.15
8	34/M	Primary	0	Normal	No	Seizure	Right temporal lobe	SX, RTx	N/A	CR	Alive	8.22
9	78/F	Primary	1	Elevated	No	Headaches	Dura of overlying cavernous sinus	SX, CTx	BR + MTX (IT) / Ara‐C (IT)	Unknown	Alive	0.34
10	30/F	Primary	0	Elevated	No	Diplopia and ptosis	Dura overlying cavernous sinus	SX, RTx	N/A	PR	Alive	1.89
11	66/F	Primary	1	Elevated	No	Left facial palsy and left hemiparesis	Right basal ganglia	SX, CTx, RTx	R + MTX	Progressive	Alive	1.76
12	50/F	Primary	0	Normal	No	Altered sensation	Dura overlying left temporal region	SX, RTx	N/A	CRu	Alive	0.41
13	61/F	Primary	0	Normal	No	Seizure, speech disturbance	Dura of left cerebral convexity	SX, RTx	N/A	CRu	Alive	1.15
14	77/M	Secondary	0	Normal	Present	Visual loss	Left optic nerve	CTx	R‐CHOP + MTX (IT)	Died	Dead	0.1
15	59/M	Secondary	0	Normal	No	Scalp swelling, dysarthria	Right frontoparietal dura	CTx	CVPR	CR	Alive	11.44
16	66/F	Secondary	3	Elevated	Present	Headaches, fevers, fatigue	Frontal lobe, occipital lobe, cerebellum	CTx	MTX (IT) + Ara‐C (IT)	PR	Dead	0.583
17	53/F	Secondary	3	Unknown	Unknown	Unknown	Left frontal lobe	RTx	N/A	PR	Dead	1.74
18	66/M	Secondary	0	Elevated	No	Visual changes	Right posterior parieto‐occipital lobe	SX, RTx	N/A	Died	Dead	0.16
19	67/F	Secondary	0	Normal	No	Unknown	Unknown	Unknown	N/A	Unknown	Alive	3.01
20	67/M	Secondary	1	Normal	No	Unknown	Unknown	Unknown	N/A	PR	Alive	1.34
21	46/F	Secondary	0	Normal	No	Unknown	Unknown	CTx	FDR + Ara‐C	CRu	Alive	3.45
22	57/F	Secondary	0	Unknown	No	Personality change, unsteadiness, memory loss	Surrounding lateral and third ventricles	CTx	IFO + Ara‐C + MTX	Unknown	Alive	Unknown
23	34/M	Secondary	0	Elevated	No	Altered sensation	Dural based sacral spinal canal	SX, CTx, RTx	R‐CHOP	CR	Alive	4.22
24	62/M	Secondary	1	Normal	No	Headaches, pain in V2 distribution of trigeminal nerve	Dura overlying optic nerves, pterygopalatine fossa, cavernous sinus, optic chiasm	SX, CTx	R	Progressive	Alive	1.57
25	69/F	Secondary	1	Elevated	No	Visual changes	Dura of right orbit and skull base	SX, RTx	N/A	Progressive	Alive	1.6
26	60/F	Secondary	1	Normal	No	Altered sensation	Skull base leptomeinges and extradural space C2‐C4	SX, CTx	Ibrutinib + R	Unknown	Alive	0.09

Abbreviations: Ara‐C, cytarabine; BR, bendamustine rituximab; BSx, B symptoms; CR complete response; Cru, complete response unconfirmed; CTx, chemotherapy; CVPR, cyclophosphamide, vincristine, prednisolone, rituximab; F, female; FDR, fludarabine; IFO, ifosfamide; IT, intrathecal; M, male; MTX, methotrexate; N/A, not applicable; OS, overall survival; PR, partial response; Progressive, progressive disease; R, rituximab; R‐CHOP, rituximab, cyclophosphamide, vincristine, doxorubicin, prednisolone; RTx, radiotherapy; SX, surgery.

Most patients with primary disease had stage I disease (n = 11) at time of original diagnosis. One patient with primary disease had MZL invading the skull and temporalis muscle and was classified as stage IV disease. Most patients with secondary MZL had stage IV disease (n = 11) at time of original diagnosis. The most common anatomical location of MZL was disease isolated to dura (50%). This was also the most common site of disease for those with primary CNS MZL (n = 9, 69%). For those with secondary CNS involvement; four (31%) had disease limited to the dura, whereas two (15%) had parenchymal only disease, one (4%) had disease limited to the leptomeninges, two (15%) had parenchymal + leptomeningeal disease, and one (4%) had leptomeningeal + optic nerve involvement. Data were not available for disease location for one patient with primary and three with secondary CNS involvement.

A minority (n = 7, 27%) of patients presented with elevated serum LDH. All patients, for whom data were available (n = 19), were negative for HIV, Hepatitis B and C. With respect to investigations, lumbar puncture was performed in 20 cases (77%) and lymphoma cells were detected in the cerebrospinal fluid in 3/20 (15%) using cytology and flow cytometry. Positron emission tomography (PET) scan was performed in 18 cases (69%), for which information was available. Magnetic resonance imaging (MRI) of the head was performed in all cases for which information was available (n = 22) and 20 patients (77%) had unifocal disease, whereas two (8%) patients, both with secondary CNS MZL, had multifocal disease. Of patients with secondary CNS MZL, six (46%) had evidence of bone marrow involvement at time of documented CNS dissemination.

Treatment was at the discretion of the treating physician and is outlined in Table 1. Overall the most common treatment was surgery + radiotherapy (n = 7, 27%). For primary disease, the most common treatment modality was surgery + radiotherapy (n = 5). Treatment data were not known for one patient with primary disease. With respect to the type of surgery performed, those with dural limited disease had complete resection and those with parenchymal disease had craniotomy and biopsy. Seven patients with primary disease that received systemic therapy were treated with a combination of methotrexate and/or rituximab (either alone or in combination with cyclophosphamide, doxorubicin, vincristine, prednisolone [R‐CHOP]). One patient was treated with bendamustine, rituximab, and alternating intrathecal cytarabine and intrathecal methotrexate. No patient with primary disease was treated with radiotherapy or chemotherapy alone. Table 2 outlines the specific treatment for each patient.

Of the 13 patients with secondary CNS MZL, eight (62%) had CNS involvement at the time of initial diagnosis of MZL. Of the remaining five cases who developed CNS involvement at a later stage, four had received treatment prior to diagnosis of CNS. The most common treatment modality for secondary disease was chemotherapy alone (n = 5). Methotrexate, rituximab, and cytarabine were used in various combinations for those with secondary disease. Other combinations included R‐CHOP, fludarabine + cytarabine, ifosfamide + cytarabine +methotrexate, and ibrutinib + rituximab.

For patients with primary CNS MZL, complete CNS response was achieved in 10 (77%) of 13 patients. No patient with primary disease had systemic progression. For patients with secondary disease, only three (23%) of 13 achieved complete CNS response. Two of these patients were treated with chemotherapy alone, whereas the other was treated with all three modalities. Four patients died, all with systemically progressive secondary CNS MZL. One died from an acute on chronic subdural hemorrhage complicating neutropenic sepsis, whereas the remaining three died from progressive lymphoma, one of which showed transformation to a high‐grade lymphoma. All patients with MZL localized to the dura were alive at last follow‐up. Best CNS response to treatment was unknown for four patients (1 with primary disease and 3 with secondary).

**Figure 1:**
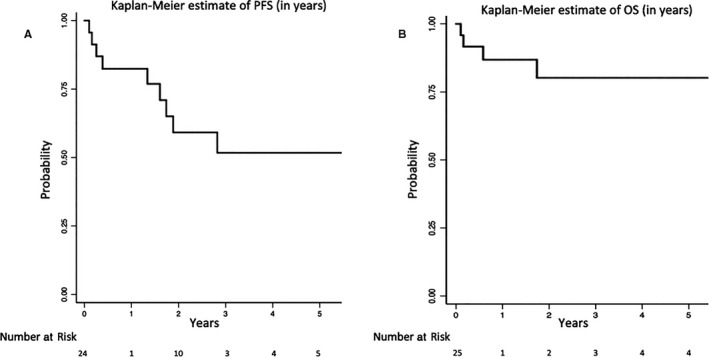
A, Kaplan‐Meir estimate of PFS (in years), B, Kaplan‐Meir estimate of OS (in years)

After a median follow‐up time of 1.9 years (range 0.1‐11.4 years), the overall median PFS was not reached with 2‐year PFS of 59% (95%CI:34%‐77%) (Figure 1A). The estimated 2‐year overall survival rate was 80% (95%CI:54%‐92%) (Figure 1B). Overall survival for primary disease at 2 years was 100%, whereas for secondary CNS MZL this was 58%.

## 4 DISCUSSION

In this series of patients with MZL, we confirm the relatively favorable outcome of primary CNS MZL and report the inferior prognosis associated with secondary CNS involvement. CNS MZL typically presents as a solitary mass occupying lesion that arises from the dura without systemic disease involvement.^3^, ^11^ It is a rare form of primary CNS lymphoma (PCNSL) and has a better prognosis compared to the more common primary CNS DLBCL.^12^, ^13^ In a recent SEER analysis of outcomes of primary CNS lymphoma by histologic subtype, the 5‐year OS rates were 30% (95%CI:28%‐32%) for DLBCL and 79% (95%CI:64%‐88%) for MZL.^12^ In this study, reported outcomes for CNS MZL were comparable with estimated 2‐year OS 80% (95%CI:54%‐92%). Patients with secondary CNS MZL appear less likely to achieve CNS complete response, and have inferior PFS and OS. There may be some factors that are associated with this inferior OS such as the advanced stage of disease at diagnosis, poor performance status, B symptoms, and experiencing a progression‐free survival event. Due to limited sample size we did not perform univariate analysis so cannot comment on the statistical significance of these associations compared to those with primary CNS MZL.

Due to the rarity of MZL, there have been no trials that evaluate different treatment options, and therefore there are no standardized treatment regimens for clinicians to utilize. There are only a few cases series of secondary MZL in the existing literature.^3^, ^14^, ^15^ With respect to treatment of primary disease, it is known that MZL is very radiosensitive and it has been suggested that patients with disease isolated to the dura may not require systemic chemotherapy.^16^ In a combined series from Memorial Sloan Kettering Cancer Center and the University of Miami, of 23 patients with dural MZL treated after biopsy or surgery, 22 achieved a complete remission.^17^ The majority of patients (87%) received cranial directed radiation therapy as a component of therapy and the 3‐year PFS was 89%.^17^ Only four patients received systemic therapy prompting the authors to conclude that complete remission is achievable with focal therapy in this indolent disease. A recent review by Chihara et al showed that localized treatment with radiotherapy ± surgery was the most common therapeutic approach for patients with primary MZL.^12^ Our data are consistent with this.

All patients with primary CNS MZL were alive at median follow‐up time, aligning with the more indolent nature of this disease. Only two patients with primary disease did not achieve complete CNS response. Given the lack of treatment experience with respect to CNS MZL, and even more so with secondary CNS disease, it is not surprising that such a range of modalities were chosen based on disease location and patient characteristics. As opposed to radiotherapy ± surgery, chemotherapy was the most common treatment modality for secondary CNS MZL, in keeping with physician selected treatment for more aggressive lymphomas.

The dura was the most common site of CNS MZL involvement, consistent with other case series.^13^ Cases of primary MZL involving the CNS parenchyma and leptomeninges are rare.^7^, ^9^, ^16^ No case of primary CNS MZL in this study had leptomeningeal involvement, but three cases involved the parenchyma including the intramedullary cord, temporal lobe and basal ganglia. The anatomic location of secondary CNS MZL was more varied and suggests that MZL with systemic involvement is more heterogenous and more likely to involve the parenchyma and leptomeninges. It is an important clinical consideration that the majority of cases of secondary CNS MZL in this study presented as unifocal disease that could easily be mistaken as a primary dural lymphoma unless thorough assessment of systemic involvement was performed.

Regarding distribution according to sex, we demonstrated a female preponderance and that this tendency may be less pronounced with respect to secondary MZL where the female to male ratio was smaller. Another difference between primary and secondary disease was the median age of diagnosis. Interestingly, there was an 11‐year difference between the median age of diagnosis for primary (51 years) and secondary (62 years) disease. Therefore, while the overall median age of diagnosis in our study (59.5 years) was similar to previously published studies, secondary involvement of CNS tissue by MZL appeared to occur in a slightly older cohort than for primary disease.^13^ This aligns with the data published by Khalil and colleagues on 8,821 cases of MZL from multiple anatomical sites that had a median age of diagnosis of 66 years.^18^ The time to CNS diagnosis was also a point of difference, however the much shorter duration of symptoms prior to detection of CNS disease in patients with secondary CNS MZL was unsurprising given these patients had a pre‐existing diagnosis of MZL or presented with widespread symptomatic disease.

There are some limitations to this study. Given the rarity of the disease, the sample size was small which limited the application of multivariable analysis. The median follow‐up time of 1.9 years could be considered short given the range over which patients were assessed. Furthermore, due to this long period of inclusion eligibility (1995‐2018), treatment modalities used may not be reflective of current practice. Finally, there were some data points that could not be collected.

## 5 CONCLUSION

MZL with secondary CNS involvement has an older age, less female predominance, more variable CNS localization and inferior prognosis relative to primary CNS MZL. Patients with primary MZL have favorable outcomes with radiotherapy. It also appears that patients with dural location of MZL have a better outcome. The optimal treatment of secondary CNS MZL remains unclear, though the use of CNS penetrating chemotherapeutics appears reasonable.

## DISCLOSURES

DV: consulting/advisory/honoraria—Roche, Janssen, Celgene, Lundbeck, Gilead, AstraZeneca, Abbvie, Seattle Genetics, Nanostring. CYC: consulting/advisory/honoraria—Roche, Janssen, Takeda, MSD, Gilead, Bristol Myers Squibb, AstraZeneca; research funding—Celgene, Roche, Abbvie; travel expenses—Roche, Amgen. MJ: consulting/advisory/honoraria—Roche, Janssen, Acerta, Gilead; research funding—Celgene, Roche, Abbvie, Janssen, Gilead. TEG: Travel funding Roche and Takeda, Employed by Roche, Basel, 1st January 2019. LJN: honoraria: Bayer, Celgene, Genentech, Gilead, Janssen, Juno, Novartis, Spectrum, TG Therapeutics. Research funding: Celgene, Genentech, Janssen, Karus Therapeutics, Merck, TG Therapeutics.

## AUTHOR CONTRIBUTIONS

AS: first draft, data compilation and analysis, creation of tables and figures. CYC: project concept, data collection and analysis, co‐writing first draft. RS, MAZ, CP, BD, JRG, LJN, DJ, GC, TEG, DV: data compilation, critical review, and final approval of final manuscript.

## Data Availability

The data that support the findings of this study are available on request from the corresponding author. The data are not publicly available due to privacy or ethical restrictions.
